# Knowledge Transfer via Pre-training for Recommendation: A Review and Prospect

**DOI:** 10.3389/fdata.2021.602071

**Published:** 2021-03-18

**Authors:** Zheni Zeng, Chaojun Xiao, Yuan Yao, Ruobing Xie, Zhiyuan Liu, Fen Lin, Leyu Lin, Maosong Sun

**Affiliations:** ^1^ Department of Computer Science and Technology Institute for Artificial Intelligence, Tsinghua University, Beijing, China; ^2^ Beijing National Research Center for Information Science and Technology, Beijing, China; ^3^ WeChat Search Application Department, Search Product Center, Shenzhen, China

**Keywords:** recommender system, pre-trained model, knowledge transfer, cross-domain transfer, cold start

## Abstract

Recommender systems aim to provide item recommendations for users and are usually faced with data sparsity problems (e.g., cold start) in real-world scenarios. Recently pre-trained models have shown their effectiveness in knowledge transfer between domains and tasks, which can potentially alleviate the data sparsity problem in recommender systems. In this survey, we first provide a review of recommender systems with pre-training. In addition, we show the benefits of pre-training to recommender systems through experiments. Finally, we discuss several promising directions for future research of recommender systems with pre-training. The source code of our experiments will be available to facilitate future research.

## 1 Introduction

With the rapid development of the Internet, users are faced with information overload, where the large quantity of online items makes it hard for users to make decisions effectively. Recommender systems aim to provide recommendations by capturing user preferences for items (e.g., movies, books, songs, news, and websites) from explicit item ratings given by users or implicit user-item interactions (e.g., browsing and purchasing histories). The application of recommender systems has enabled personalized services in many scenarios, such as e-commerce and website browsing.

Recommender systems are usually faced with data sparsity in real-world scenarios. Recommender systems can suffer when providing recommendations for new items or users due to lack of information, which is known as the cold start problem (Gope and Jain, 2017). Without sufficient data, the model parameters cannot be well estimated and users’ preference cannot be well modeled. It has been shown that the data sparsity problem in recommender systems can be alleviated by transferring knowledge from other domains or tasks (Cantador et al., 2015) and integrating heterogeneous external knowledge (Guo et al., 2020).

In the field of natural language processing, pre-trained models have achieved great success recently on a broad variety of tasks by knowledge transfer (Qiu et al., 2020). Models are usually first pre-trained on large-scale unsupervised data to learn universal language representations and then fine-tuned on downstream tasks to achieve knowledge transfer. The models can be pre-trained to learn either shallow context-free word embeddings (Mikolov et al., 2013) or deep context-aware language representations (Devlin et al., 2019). The resulting language representations have proven to be useful not only for different tasks [e.g., natural language inference and question answering (Devlin et al., 2019)] but also for different scenarios, such as few-shot learning (Brown et al., 2020) and domain adaptation (Rietzler et al., 2020).

In the context of recommender systems, we can group works that utilize pre-training mechanisms to improve the precision of recommendation into two categories: feature-based models and fine-tuning models. The feature-based models generally use pre-trained models to obtain features from side-information (e.g., the content of items and knowledge graphs) for users and items (Guo et al., 2020). The fine-tuning models leverage the user-item interaction records to pre-train a deep transferable neural model, which is subsequently fine-tuned to downstream recommendation tasks (Chen et al., 2019c). Generally, the benefits of pre-training to recommender systems can be summarized as being twofold: 1) pre-training tasks can better exploit user-item interaction data to capture user interests, and 2) pre-training can help integrate knowledge from different tasks and sources into universal user/item representations, which can be further adapted to various scenarios in recommender systems, such as cold starts and cross-domain transfer.

The contributions of this survey can be summarized the following. 1) *Systematic Review*: We provide a systematic review of the pre-training methods for recommender systems with a clear taxonomy. 2) *Empirical Results*: We present empirical results to show the benefits of pre-training to recommender systems. We conduct experiments on the task of movie recommendation where different types of knowledge are integrated by pre-training for better recommendations, especially in the cold start and cross-domain transfer scenarios. 3) *Future Directions*: Based on the review and experiments, we discuss several promising directions for future research, including how to better leverage pre-training approaches to improve recommender systems and how recommender systems can motivate better pre-training models.

The rest of the survey is organized as follows: In Sections 2 and 3, we provide a review of existing methods of recommender systems with pre-training; in Section 4, we conduct experiments to empirically show the benefits of pre-training to recommender systems; and in Section 5, we discuss promising directions for future research.

## 2 Feature-Based Models

Feature-based models leverage side-information (e.g., contents of items, knowledge graphs, and social networks) using pre-trained models to directly enrich the representations of users or items. Different from collaborative filtering (CF) methods that learn the representations from user-item interaction records (Hu et al., 2008; Su and Khoshgoftaar, 2009; Wang et al., 2020a), feature-based models focus on extracting widely applicable features from external information sources with pre-trained models and then integrate these features into the recommendation framework. By combining rich side-information and user-item interaction data, feature-based models can potentially solve some challenges, such as the data sparsity problem.

The general idea can be illustrated as follows. Given the external information resource, the pre-trained models are applied to obtain the external feature vectors, ${\hat{u}}_{i}$ and ${\hat{v}}_{j}$ for user $u_{i}$ and item $v_{j}$ respectively. Denote ${\tilde{u}}_{i}$ and ${\tilde{v}}_{j}$ as the features learned from the user-item interaction records for user $u_{i}$ and item $v_{j}$ respectively. Then the final representations $u_{i}$ and $v_{j}$ for each user and item are obtained by aggregating external feature vectors and the vectors from the user-item interaction data:

$$u_{i}=g_{u}\left({\tilde{u}}_{i},{\hat{u}}_{i}\right),\;v_{i}=g_{v}\left({\tilde{v}}_{i},{\hat{v}}_{i}\right),$$ (1)

where $g_{u}\left(\cdot \right)$ and $g_{i}\left(\cdot \right)$ are aggregate functions. The preference score for user $u_{i}$ and item $v_{j}$ is calculated by

$$s\left(u_{i},v_{j}\right)=f\left(u_{i},v_{j}\right),$$ (2)

where f(⋅) is a recommendation function, which can be factorization machines (Rendle, 2010) and deep neural networks (He et al., 2016; Fan et al., 2018) and the like.

According to the type of external information resources, feature-based pre-trained models can be roughly categorized into content-based recommendation, knowledge graph-based recommendation, and social recommendation models. Different types of meta-information require different pre-trained models. We will introduce how the pre-training mechanism is used in these three kinds of recommender systems in detail.

### 2.1 Content-Based Recommendation

Content-based recommendation assumes that users prefer items similar to those being historically interacted with. Therefore, it is important for content-based recommender systems to encode the content of items into expressive low-dimensional representations. The pre-trained models have proven to be powerful in extracting generally applicable representations from text, images, audio, etc. Hence, many works learn features from the content of items to serve recommendation models.

Liang et al. (2015) pre-train a multilayer neural network to extract audio features for music recommendation via a semantic tag prediction task. In terms of dealing with textual data for recommendation, such as reviews (Zheng et al., 2017), tweets (Gong and Zhang, 2016), and news (Cao et al., 2017), pre-trained word embeddings or pre-trained sentence encoders become indispensable. Some works simply use the average of word embeddings to represent the whole documents (Brochier, 2019; Cenikj and Gievska, 2020). Other works focus on the design of task-specific frameworks, where the input word embeddings will be fed into a complex document encoder to generate the document representation (Song et al., 2016; Tan et al., 2016; Xu et al., 2016; Nguyen et al., 2017; Zhang et al., 2017). Similarly, the pre-training mechanism is widely used in image feature extraction for recommender systems (Chu and Tsai, 2017; He and McAuley, 2016a, He and McAuley, 2016b).

### 2.2 Knowledge Graph-Based Recommendation

Knowledge graph-based recommendation introduces knowledge graphs (KGs) as side-information to better characterize users and items. A KG is a structured graph containing fruitful facts and connections between users, items, and other related entities. Amounts of side-information, such as the user profiles, the attributes of items, and relations between cross-domain items, can be integrated into KGs. Hence, KGs can help recommender systems to capture essential knowledge and provide explanations for the recommendation results.

Various KGs have been used in different works. For instance, some works construct knowledge graphs with items and their related attributes (Zhang et al., 2016; Wang et al., 2018b; Huang et al., 2018). Some other works add users to build user-item graphs, which contain information, including the item attributes, user behaviors (e.g., purchase, click), and user profiles (Wang et al., 2018a; Cao et al., 2019; Dadoun et al., 2019). With the informative heterogeneous user-item graphs, the potential relations between users and items can be modeled directly.

In order to exploit KGs, one line of KG-based methods seeks to encode the KG into low-dimensional pre-trained embeddings with the knowledge graph embedding (KGE) methods (Bordes et al., 2013; Wang et al., 2014; Lin et al., 2015; Ji et al., 2015; Trouillon et al., 2016; Shi and Weninger, 2017; Balazevic et al., 2019). Then as stated in Eq. 1, the knowledge graph embeddings are aggregated with user/item features obtained from interaction data. Experimental results show that KGs are powerful information resources and can improve the performance of recommendation significantly (Qin et al., 2020).

### 2.3 Social Recommendation

Social recommendation is a type of recommendation method that utilize online social relations as an additional input (Tang et al., 2013). Different from KGs, which integrate various information about users and items, social graphs focus on modeling the social relation between users. Homophily theory indicates that the preference of a user is similar to or influenced by their socially connected friends (McPherson et al., 2001). Similar to the KG-based recommendation, many social recommender systems seek to integrate the pre-trained social network embeddings, which indicates the degree that a user is influenced by his/her friends (Guo et al., 2018; Wen et al., 2018; Zhang et al., 2018; Sathish et al., 2019; Chen et al., 2019a, Chen et al., 2019b).

### 2.4 Summary

Feature-based models preprocess side-information with various pre-trained models to obtain the embedding of users or items, which are then integrated into the recommender systems. By utilizing side-information, feature-based approaches are able to construct expressive representations for users and items and can achieve significant improvement for recommendation. In addition to have side-information, exploiting large-scale interaction data is also crucial to recommender systems. Therefore, some recent efforts have been made to pre-train models with user-item interaction records, which are introduced in the following section.

## 3 Fine-Tuning Models

The fine-tuning models for recommendation first pre-train the parameters with large-scale interaction data. The models are then transferred to downstream tasks by simply fine-tuning the pre-trained parameters. The fine-tuning paradigm has shown the effectiveness in other areas, such as natural language processing (Devlin et al., 2019; Joshi et al., 2020). According to the model architecture, we can categorize existing works in recommender systems into two classes: shallow neural networks (Hu et al., 2018; Ni et al., 2018) and deep residual neural networks. Existing deep residual neural networks for recommendation can be further divided into BERT-based models (Chen et al., 2019c; Sun et al., 2019; Yang et al., 2019) and parameter-efficient pre-trained convolutional neural networks (Yuan et al., 2020).

### 3.1 Shallow Neural Networks

Early works attempt to achieve knowledge transfer with shallow neural networks as base models, such as shallow multilayer perceptrons (MLP), and recurrent neural networks. Hu et al. (2018) attempt to improve recommendation via cross-domain knowledge sharing. They conduct a baseline experiment where an MLP together with user and items embeddings is pre-trained on the source domain. The user embeddings are then transferred to the target domain as warm-up. Experimental results show that this simple method cannot achieve obvious improvement in recommendation performance. The results demonstrate that the model architecture and pre-training tasks need to be carefully designed to achieve effective knowledge transfer in fine-tuning models. Therefore, many efforts have been devoted to investigating efficient pre-training tasks and transferrable model architectures.

Ni et al. (2018) propose the DUPN model which is able to learn universal user representations across multiple recommendation tasks. DUPN takes interaction sequence as inputs and then applies LSTM and an attention layer to obtain the user representations. DUPN is pre-trained by multiple tasks objectives, including click-through rate prediction, shop preference prediction, and price preference prediction. Experimental results show that DUPN can achieve not only improvement on the tasks used for pre-training but also faster convergence and promising results in related new tasks. Though the user representations learned with DUPN are powerful, DUPN requires many extra information sources like user profiles to facilitate different pre-training tasks.

### 3.2 BERT-Based Models

In order to capture the dynamic user preference, many researchers attempt to exploit the user chronological interaction sequence, which is called the session-based recommendation. Similar to natural language processing (NLP) that targets on word sequence, session-based recommendation investigates the item sequence and aims to take sequential information into account. Inspired by the rapid progress in pre-trained language models in NLP (Devlin et al., 2019; Liu et al., 2019; Joshi et al., 2020), many efforts have been devoted to capturing information from the user behavior sequence with pre-trained models, especially BERT-based models. In this section, we introduce the pre-trained BERT-based models for recommendation, including the widely used masked item prediction task, the architecture of BERT, and advanced BERT-based models for recommendation.

#### 3.2.1 Masked Item Prediction

Similar to the masked language modeling task in NLP, the masked item prediction task (MIP) is proposed and widely applied in many recommender systems. In the MIP task, given the interaction sequence, some of the items are randomly masked. The models are required to reconstruct the masked item. Formally, we denote the interaction sequence in chronological order for user *u* as ${\text{s}}_{u}=\left\{v_{1},v_{2},\ldots ,v_{N}\right\}$, where $v_{i}$ is the item that *u* interacted with at time step *i*. For pre-training stage, some input items in the interaction sequence are randomly masked with special token [MASK]. Then the models are asked to predict the masked items:

$$Inputs:\;\left\{v_{1},v_{2},v_{3},v_{4},v_{5}\right\}\to \left\{v_{1},v_{2},{\left[MASK\right]}_{1},v_{4},{\left[MASK\right]}_{2}\right\},$$

$$Labels:\;\;{\left[MASK\right]}_{1}=v_{3},\;\;{\left[MASK\right]}_{2}=v_{5}.$$

The objective of this task is the negative likelihood of the masked targets. Unlike the left-to-right Next Item Prediction (NIP) task that is used in many session-based recommender systems (Yu et al., 2016; Wu et al., 2017; Hidasi and Karatzoglou, 2018), MIP enables the models to learn representations of user behavior sequences from the whole context. Moreover, the MIP task can overcome the limitation that a rigidly ordered sequence is not always practical in user behaviors (Sun et al., 2019). Therefore, models pre-trained with the MIP task can achieve promising results.

#### 3.2.2 BERT for Recommender System

**FIGURE 1:**
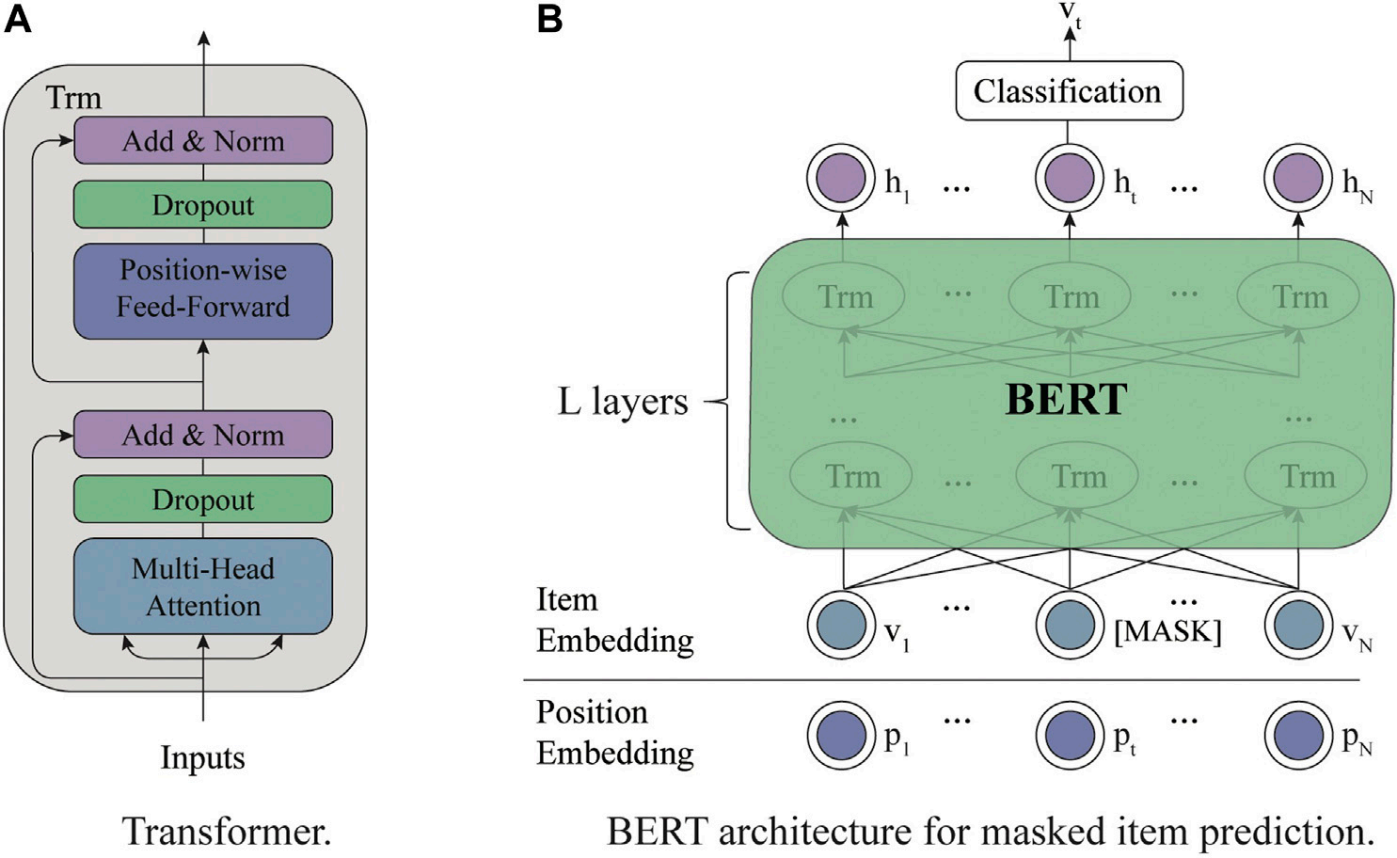
**(A)** is the framework of Transformer. **(B)** is the overall architecture of BERT for masked item prediction task.

Inspired by the success of BERT (Devlin et al., 2019) in text understanding, many researchers adopt BERT for recommendation. In this section, we will introduce the architecture of BERT and how to utilize BERT for recommender systems. For convenience, we denote the BERT model pre-trained with the MIP task as BERT4RS. As shown in Figure 1, BERT is based on a multilayer bidirectional Transformer (Vaswani et al., 2017). The Transformer contains two sublayers: multihead attention sublayer and point-wise feed-forward network.

- *Multihead Attention*: an attention mechanism has been used successfully in various sequence modeling tasks, which enables models to focus on important information. The attention function takes queries $Q\in {\mathbb{R}}^{l_{Q}\times d_{k}}$, keys $K\in {\mathbb{R}}^{l_{K}\times d_{k}}$, and values $V\in {\mathbb{R}}^{l_{K}\times d_{v}}$ as inputs and computes the outputs as follows, where $d_{k}$ and $d_{v}$ are the dimensions and $l_{Q}$ and $l_{K}$ are the length of sequences.

$$Attention\left(Q,K,V\right)=soft\max\left(\frac{QK^{T}}{\sqrt{d_{k}}}\right)V.$$ (3)

Self-attention is a special attention function aiming to learn the representation for a single sequence, in which the input sequence performs as the queries, keys, and values. Instead of performing a single attention function, Transformer employs the multihead self-attention function, which allows the model to jointly attend to information from different vector subspaces. Specifically, this mechanism first linearly projects the input sequence into *h* subspaces and then produces the output representation with *h* attention functions.

$$MultiHead\left(H\right)=Concat\left(head_{1},head_{2},\ldots ,head_{h}\right)W^{O},$$ (4)

$$head_{i}=Attention\left(HW_{i}^{Q},HW_{i}^{K},HW_{i}^{V}\right),$$ (5)

where $H\in {\mathbb{R}}^{l\times d_{o}}$ is the input sequence, and $W_{i}^{Q}\in {\mathbb{R}}^{d_{o}\times d_{k}}$, $W_{i}^{K}\in {\mathbb{R}}^{d_{o}\times d_{k}}$, $W_{i}^{V}\in {\mathbb{R}}^{d_{o}\times d_{v}}$, and $W^{O}\in {\mathbb{R}}^{hd_{v}\times d_{o}}$ are learnable parameter matrices.

- *Point-wise Feed-Forward Network*: the multihead attention function enables the model to integrate information from different positions with linear combinations. Then the point-wise feed-forward network endows the model nonlinearity. In this sublayer, a fully connected feed-forward network is applied to each position separately and identically.

FFN(x)=ReLU(xW1+b1)W2+b2. (6)

The sublayer consists of two linear transformations and a ReLU activation. It should be noted that though the transformations are the same across different positions, the parameters are different for different layers.

Following each sublayer, a residual connection (He et al., 2016) and a layer normalization operation (Ba et al., 2016) are employed for stabilizing and accelerating the network training. As shown in Figure 1B, after *L* layers of Transformer, the final hidden states of masked tokens are fed into a feedforward network to get the output distribution over target items.

BERT4RS is effective in modeling user preference from the historical behaviors. Sun et al. (2019) train a two-layer BERT with the MIP task, which achieves the state-of-the-art performance on the session-based next item recommendation task. They observe that both the BERT architecture and the MIP task can significantly improve the performance, and stacking multiple Transformer layers can further boost the performance on large-scale datasets, which provides fundamental support for the following models.

Chen et al. (2019c) proposed to fine-tune BERT4RS with a content-based click through prediction task. Specifically, the user representation u is produced with pre-trained BERT from the historical behavior sequence, and the item representation v is extracted from its content. Then the preference score is calculated with Eq. 2, where f(⋅) is an MLP layer.

Some downstream recommendation tasks, such as next basket recommendation (Rendle et al., 2010; Yu et al., 2016) and list-wise recommendation (Shi et al., 2010; Zhao et al., 2017), require the model to capture the relations between item sequences. Therefore, in addition to MIP, researchers propose some sequence-level pre-training tasks to pre-train the model. Yang et al. (2019) adopts the BERT4RS for next basket recommendation task, which is pre-trained with MIP and next basket prediction (NBP) tasks. In real-world scenarios, a user usually buys or browses a series of items (a basket) at a time. Given two baskets, NBP requires the model to predict whether the two baskets are adjacent in the purchase records:

$$Inputs:\;\;\left\{\left[CLS\right],\;v_{1}^{1},\;\;\;v_{2}^{1},\;\;v_{3}^{1},\;\;\left[SEP\right],\;\;v_{1}^{2},\;\;v_{2}^{2},\;\;v_{3}^{2},\;\;\;\left[SEP\right]\right\}$$

$$\to \;\;\left\{\left[CLS\right],\;v_{1}^{1},{\left[MASK\right]}_{1},v_{3}^{1},\;\;\left[SEP\right],\;\;v_{1}^{2},\;\;v_{2}^{2},{\left[MASK\right]}_{2},\left[SEP\right]\right\}$$

$$MIP\;Lables:\;{\left[MASK\right]}_{1}=v_{2}^{1},\;\;{\left[MASK\right]}_{2}=v_{3}^{2}$$

NBP Lables: IsNext/NotNext,

where $v_{i}^{1}$ and $v_{i}^{2}$ are items from different baskets and [CLS] and [SEP] are special tokens. The final hidden state of [CLS] is used to predict the NBP label.

### 3.3 Parameter-Efficient Pre-trained Model

The pre-training mechanism can enable models to capture user preference from behavior history via self-supervised learning. Experimental results show that it can achieve significant improvement for recommendation. However, fine-tuning models separately for different tasks is computationally expensive and memory intensive (Mudrakarta et al., 2018; Stickland and Murray, 2019), especially for resource-limited devices.

**FIGURE 2:**
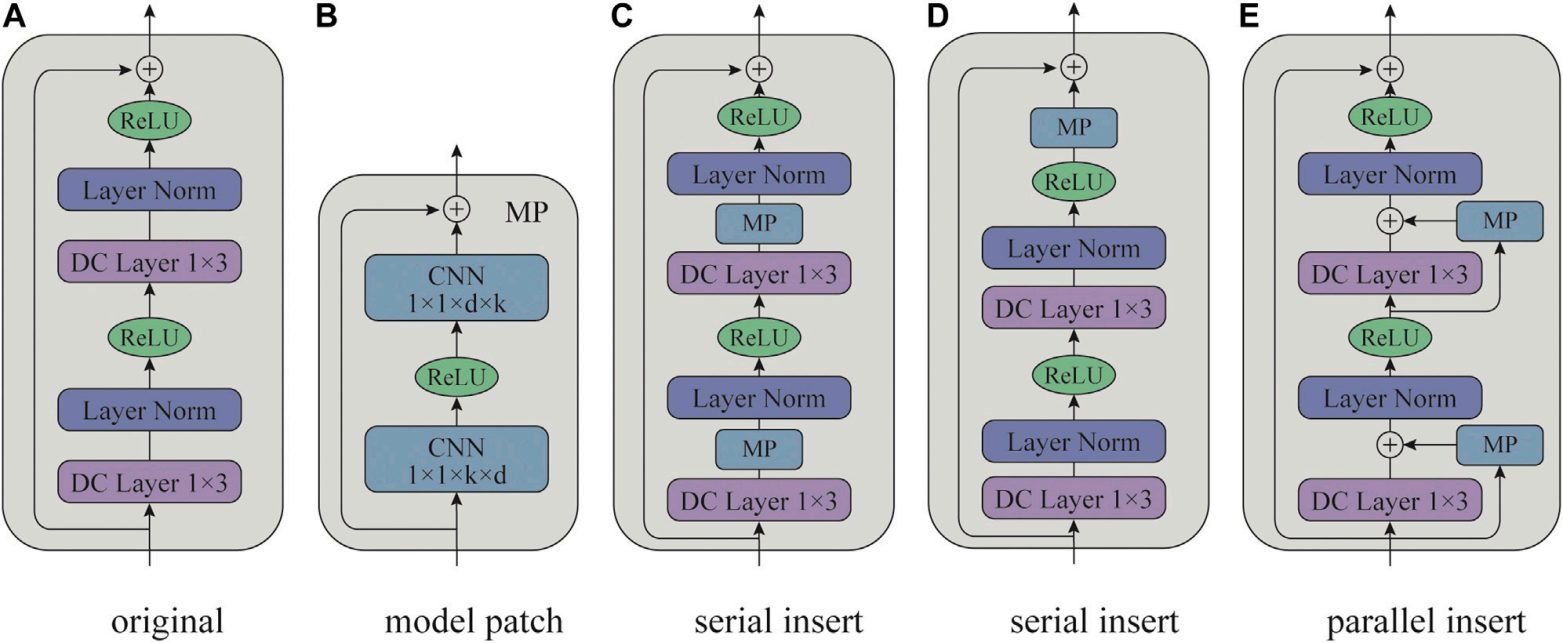
The model architecture of Peterrec (Yuan et al., 2020). **(A)** is the original residual block of the pre-trained model. **(B)** is the model patch (MP) block. **(C–E)** are different approaches to insert MP blocks for fine-tuning. ⊕ indicates element-wise addition operation. 1×3 and 1×1×d×k refer to the kernel size of convolutional layers.

To address this issue, Yuan et al. (2020) proposed the Peterrec, which utilizes a grafting neural network in fine-tuning, termed as model patch (MP). By inserting MPs in the pre-trained models, the fine-tuning networks can keep all pre-trained parameters unchanged. As shown in Figure 2, Peterrec is a stack of dilated convolutional (DC) layers (Yu and Koltun, 2016), and there is a residual connection between every two DC layers. Similar to other pre-trained models, Peterrec employs the MIP task to pre-train. During the fine-tuning stage, the MP blocks, which are simple residual two-layer convolutional networks, are inserted around the original DC layers. The pre-trained parameters are shared across different tasks. Only parameters of the MP blocks will be fine-tuned. To accelerate fine-tuning and minimize the number of parameters, the MP blocks are designed in a bottleneck architecture. Specifically, the first convolutional layer projects the *k* dimensional channels into *d* (d≪k) dimensional vectors, and the second layer projects it back to its original dimension. Thus, the number of inserted parameters can be less than 10% parameters of original pre-trained models. Empirical results show that the pre-trained Peterrec is useful for various downstream tasks, including user profile prediction and top-k recommendation. Moreover, it can achieve promising results even when the user is cold in the new target domain, which proves the effectiveness of pre-trained models in knowledge transfer for recommendation.

### 3.4 Summary

The pre-training mechanism works well in many recommendation tasks. Early works explore pre-training with shallow neural networks. Inspired by the success of pre-trained language models in NLP, the deep residual models pre-trained with MIP task are widely used in many different recommender systems. Although it has been proven effective to employ pre-trained models for recommendation, there are many open challenges to be addressed, which will be discussed in Section 5.

## 4 Experiment of Recommender System With Pre-training

In this section, we conduct experiments to verify the benefits of pre-training to recommender systems. We take next-item and rating prediction recommender systems as examples and investigate the potential of pre-trained recommender systems in cold start problem and cross-domain knowledge transfer.

### 4.1 Dataset

We evaluate the models on MovieLens^1^, which is a representative and popular real-world benchmark in recommendation field. We choose the well-established lightweight version MovieLens 1 m (ML-1m). Each user-item interaction contains a timestamp and an integer rating score from 1 to 5. We follow Tang and Wang (2018) for data preprocessing. Users and items with too few ratings (<5) are filtered out. We leave the last 5 items of each sequence to form the validation set (2 items for each user) and the test set (3 items for each user). To investigate the effectiveness of pre-trained recommender systems in cross-domain context, we divide all the movies in ML-1m into two domains according to movie genres and obtain interaction sequences of the same users on two domains. The scale of the target domain is smaller than the source domain (ML-1m-src). To better evaluate the models under the cold start scenario, we further truncate the interaction sequences in the target domain such that the sequence length of each user is less than 10, which results in the ML-1m-tgt set.

Apart from user-item interactions, we also use the meta information from IMDB^2^ (IMDB 5000 Movie Dataset) to provide side-information for the items. For each movie, we query the director and three main actors. The statistics of the processed datasets are summarized in Table 1.

**TABLE 1:** Statistics of datasets. #User, #Item, #ItMeta, and #InA indicate the number of users, items, items with meta information, and interactions, respectively. AvgLen refers to the average length of input training sequences.

Dataset	#User	#Item	#ItMeta	#InA	AvgLen
ML-1m	6,040	3,706	1,987	999k	165.7
ML-1m-src	6,040	2,656	1,381	622k	97.9
ML-1m-tgt	6,040	1,012	512	81k	8.85

### 4.2 Task Settings and Baselines

We regard user ratings as interaction sequence based on timestamps and evaluate the models on two representative recommendation tasks, including NIP and rating prediction. For both tasks, models are first pre-trained on ML-1m-src and then fine-tuned and evaluated on ML-1m-tgt.

Next Item Prediction: NIP aims to predict the next item that is likely to interest the user given the historical interaction sequence. For each sequence, we generate negative samples in which items are not seen in training data. Since movie rating behavior is not strictly sequential, we treat the three items in each test sequence as equivalent. We adopt Normalized Discounted Cumulative Gain (NDCG@K) and Recall@K as evaluation metrics. Models are provided with IMDB 5000 as side-information for items.

For baseline methods, we choose two representative models. Caser (Tang and Wang, 2018) is a shallow recommendation model which combines vertical and horizontal CNNs to extract user and item representations. BERT4Rec (Sun et al., 2019) utilizes BERT as architecture backbone for session-based recommendation. To investigate the potential of pre-training in deep recommendation models, we increase the number of layers in BERT4Rec.

*Rating Prediction.* We also evaluate the pre-trained recommender systems on the rating prediction task, where models are required to predict the rating of the items based on users’ historical feedback and profile. Following previous works in rating prediction (Zheng et al., 2016; Xing et al., 2019), we formalize the rating prediction task as a regression task and adopt Root Mean Square Error (RMSE) and Mean Absolute Error (MAE) as evaluation metrics. In addition to BERT4Rec (Sun et al., 2019), we choose DACF (Kuchaiev and Ginsburg, 2017) which utilizes deep autoencoder for rating prediction as our baseline models.

### 4.3 Model Design Choices

We investigate the effect of different design choices on pre-trained recommendation systems, including knowledge transfer approach and pre-training tasks. We also explore the potential of pre-trained recommendation systems in integrating external side-information.

*Knowledge Transfer Approach.* Our main purpose is to verify the effectiveness of pre-training on recommender systems. Therefore, we first investigate pre-training session-based recommendation models on ML-1m-src before fine-tuning on ML-1m-tgt. We hope that the knowledge learned from pre-training can be transferred between domains. During fine-tuning, we investigate two approaches to knowledge transfer: 1) embedding transfer where we can transfer and fine-tune only the input embedding layer (i.e., user embeddings and external knowledge embeddings) and leave other layers randomly initialized and learned from scratch in the target domain and 2) model transfer where we also explore transferring and fine-tuning the whole model on the target domain. In this way, user-item interaction knowledge is also expected to be transferred^3^.

*Pre-training Tasks*: the design of pre-training tasks is crucial for pre-trained models to capture general knowledge from large-scale data. We compare two widely used pre-training tasks in recommender systems: 1) NIP recurrently predicts the next item in a left-to-right fashion, and 2) Masked Item Prediction (MIP), which randomly masks the items and predicts the masked item according to bidirectional context.

*External Knowledge*: external knowledge is shown to be effective in handling data sparsity problem in recommender systems. We investigate whether external knowledge can be combined with general knowledge learned from pre-training to achieve better results. Specifically, we constrain the item features by concatenating the external knowledge embeddings (i.e., director embeddings and actor embeddings) with the item embeddings to obtain external knowledge enhanced item embeddings.

### 4.4 Implementation Details

We find the optimal settings of hyperparameters via grid search on the validation set.

For the Caser model, we use implementation provided by the authors^4^. Batch size is searched from {128,256,512,1024} and tuned as 512. Hidden dimension size is searched from {30,50,100,150} and tuned as 100. We employ Adam to optimize the model, and we set the learning rate as 1e−3, weight decay as 1e−6. For masked item prediction, we set the mask probability as 0.2. Following Devlin et al. (2019), we do not always replace the masked item with [MASK] token. If an item is chosen, we replace it with 1) [MASK] 80% of the time, 2) a random item 10% of the time, and 3) the unchanged item 10% of the time.

For BERT4Rec (Sun et al., 2019) model, we choose a PyTorch implementation^5^. We keep most of the original hyperparameter and initialization strategy settings. Batch size is searched from {128,256,512} and tuned as 256. Hidden dimension size is used to search {64,128,256} and is tuned to 128. We employ Adam to optimize the model, and we set the learning rate as 1e−3. We add user embedding and set the user embedding size as 32. We change the layer of BERT blocks to 6. For masked item prediction, we set the mask probability as 0.15 and use the same masking strategy as in Caser.

When incorporating external knowledge, the size of director and main actor embedding is 25 for Caser and 16 for BERT4Rec. The number of negative samples is set to 100. In evaluation, negative samples size is 100 for both models.

For rating prediction task, the rating scores are regarded as classification tags, and we simply concatenate rating embedding for each user-item interaction and set the embedding size to 128. The output layer of BERT4Rec is modified from classification to regression. The model is required to predict the rating scores for rating-masked items during training and the last three items of each user sequence during testing. For collaborative filtering model, we choose a deep learning implementation DACF^6^ and keep the default settings.

### 4.5 Experiment Results

The experiment results on ML-1m-tgt are reported in Table 2 and Table 3, from which we have the following observations^7^:

1) Pre-training boosts the recommendation performance for both models. However, the effects of knowledge transfer approaches are correlated with model capacity. Specifically, for the shallow Caser model, embedding transfer (i.e., only transfer the embedding layer) tends to achieve larger improvements with model transfer (i.e., transfer the whole model). In contrast, model transfer achieves much better performance for deep BERT4Rec model. We hypothesize that general knowledge about user-item interactions can be better captured by high-capacity models with pre-training, leading to better performance in transferring whole models.
2) For pre-training tasks, masked item prediction achieves better performance than NIP for BERT-based models, which is consistent with the results reported by Sun et al. (2019). One possible reason is that movie rating behavior does not strictly follow chronological order (i.e., the chronological order of two items are likely to be swapped). Therefore, by integrating bidirectional information, masked item prediction can learn better representations during pre-training. However, the superiority of masked item prediction does not seem obvious on Caser. It is probably because masked item prediction creates gap between pre-training and fine-tuning (e.g., prediction during fine-tuning is based on unidirectional context), which cannot be easily overcome by shallow models.
3) Incorporating external knowledge improves the performance of pre-trained BERT4Rec model. We note that for pre-trained Caser, the effect of external knowledge is not significant and sometimes even negative. We speculate that the limited capacity of simple models hinders the integration of external knowledge. Besides, simple concatenation cannot well integrate external knowledge with general knowledge learned from pre-training either. More advanced methods can be developed to inject external knowledge into pre-trained recommendation models.
4) Pre-training also improves the performance of the recommender system in rating prediction task. The two models both achieve lower error scores when first pre-trained on ML-1m-src. However, since the rating value of an item barely depends on sequential information, DACF outperforms BERT4Rec, which is designed for sequential recommendation. Most existing fine-tuning models can be only applied with sequential data.

**TABLE 2:** Performance (%) comparison of different settings on NIP of ML-1m-tgt.

Model	Transfer	Task		With meta	Test result					
		NIP	MIP		NDCG@3	NDCG@5	NDCG@10	Recall@3	Recall@5	Recall@10
Caser	None				14.46	16.82	19.83	13.20	17.30	23.91
				✓	14.34	16.76	19.73	13.10	17.27	23.82
	Embedding	✓			14.70	16.92	19.89	13.39	17.24	23.79
				✓	14.41	16.76	19.62	13.35	17.43	23.72
				✓		15.08	17.25	20.02	13.74	17.49	23.59
				✓	15.40	17.62	20.39	14.01	17.85	23.93
	Model	✓			15.23	17.41	20.36	13.57	17.34	23.82
				✓	12.00	14.62	17.88	11.44	15.97	23.14
					✓		15.03	17.19	19.94	13.73	17.47	23.54
					✓	11.88	14.69	17.93	11.47	16.33	23.43
	BERT4Rec	None				42.34	51.56	61.18	40.94	56.79	77.87
					✓	43.92	53.03	62.41	42.31	58.03	78.58
Embedding		✓			42.95	52.09	61.61	41.46	57.25	78.10
				✓	43.95	53.11	62.66	42.38	58.17	79.08
				✓		43.82	53.22	62.74	42.53	58.76	79.61
				✓	44.89	54.35	63.84	43.33	59.65	80.41
Model		✓			43.75	52.84	62.20	42.28	57.95	78.48
				✓	44.58	53.59	63.03	42.93	58.49	79.15
					✓		44.58	54.13	63.71	43.31	59.78	80.75
					✓	45.12	54.48	63.97	43.72	59.88	80.64

**TABLE 3:** Performance (%) comparison of different settings on rating prediction of ML-1m-tgt.

Model	RMSE	MAE
DACF	0.987	0.867
DACF transfer	0.976	0.859
BERT4Rec	1.371	0.960
BERT4Rec transfer	1.261	0.903

In conclusion, recommender systems could benefit from pre-training, which effectively transfer knowledge between domains and tasks, and have potential in solving problems, including cold start. It is worth exploring design models to utilize diverse data for various downstream tasks.

## 5 Open Challenges and Future Directions

Although the pre-trained models have shown their power in recommender systems, the challenges still exist. In this section, we suggest five open challenges and future directions of pre-trained models in recommendation, where (1), (2), and (3) are discussion about how to better utilize pre-trained models in various recommendation scenarios and (4) and (5) mainly focus on how to improve the pre-training mechanism to better serve recommender systems.

1) *Cold Start*: collaborative filtering recommender systems rely heavily on user historical behavior data and can suffer from the cold start problem. To alleviate the problem, some approaches (Ma et al., 2011; Manotumruksa et al., 2016; Yu et al., 2018) use side-information, such as user profile and item attributes, to enrich user/item representations. Besides, there are some efforts utilizing more efficient learning mechanism to alleviate the heavy data reliance, such as few-shot learning (Lee et al., 2019; Li et al., 2019).

Pre‐trained language models can significantly improve few-shot performance (Brown et al., 2020) in NLP tasks. Similarly, in terms of recommendation, pre-trained models can be applied for cold start problem by learning transferable representations of the shared information between large-scale general domain and sparse target domain. For example, if a user is cold in the target domain, it is useful to transfer his/her representation pre-trained in the general domain; if an item is cold, its representation can be estimated by leveraging the pre-trained representations of external information. Peterrec (Yuan et al., 2020) is a good exploration which achieves user cold start with pre-trained models.

2) *Knowledge Enhanced Pre-training*: knowledge graphs can provide rich domain knowledge, world knowledge, and commonsense knowledge for recommendation. Therefore, by incorporating KGs into recommendation, user preference and relations between items can be captured more accurately. Many KG-based approaches are proposed recently and have achieved promising results (Zhang et al., 2016; Wang et al., 2018b; Tang et al., 2019; Qin et al., 2020). However, few works consider directly injecting external structured knowledge into pre-trained models for recommendation.

In fact, many knowledge-enhanced pre-trained language models (Zhang et al., 2019; Liu et al., 2020; Wang et al., 2020b) have shown that fusing the structured knowledge into pre-trained models can significantly boost the performance of original models. Knowledge information can help models better characterize users and items and thus can improve the performance of recommendation.

3) *Social Relation Enhanced Pre-training*: social relations provide a possible perspective for personalized recommendation. Users who are connected are more likely to share similar preferences. Pre-trained models are proficient in capturing user interest from their historical interaction records. Therefore, the social relations between users can be viewed as meta-relations between user-item interaction sequences; i.e., the interaction sequences of closely connected users are encouraged to share similar representations. Based on this, sequence-level pre-training tasks can be proposed to help models to generate more expressive user/item representations.

Another possible direction is to employ social relation enhanced pre-trained models to solve user cold start problem. Social relations can provide clues for the user interest. However, it is still challenging to make full use of the rich information contained in the neighboring users in social relation graphs during pre-training.

4) *Pre-training Tasks*: currently, all the deep fine-tuning approaches rely on the MIP task to pre-train the model. And these works focus on extracting user interest from their historical sequential records. However, limited by the computing ability and memory of GPUs, only the most recent interaction records that represent recent user preference can be utilized by recommendation models. Besides, MIP can only utilize sequential data, while rich heterogeneous information is usually available in many real-world scenarios. Therefore, designing new self-supervised pre-training tasks is important to make full use of the large-scale heterogeneous data for recommendation.
5) *Model Architecture and Model Compression*: the pre-trained models are effective in various recommendation tasks. However, their high computation complexity makes it hard to deploy them in real-world scenarios. To address the problem, it would be helpful to perform model compression (McCarley, 2019; Gordon et al., 2020) or improve the model architecture. Besides, fine-tuning separately for each down-stream task is quite time consuming and memory intensive. The model patch (Yuan et al., 2020) is a good attempt to reduce memory cost. However, it is still an urgent need to achieve fast and effective knowledge transfer from pre-trained models to multiple down-stream tasks.

## 6 Conclusion

In this article, we investigate pre-trained models for recommendation and summarize the efforts devoted to this direction. We conduct a comprehensive overview of two types of pre-trained models for recommendation, including feature-based models and fine-tuning models. Then we conduct experiments to show the benefits of pre-training for recommender systems. Finally, open challenges and future direction are discussed, hoping to promote the progress of this domain.

## Footnotes

1
https://grouplens.org/datasets/movielens/

2
https://www.kaggle.com/carolzhangdc/imdb-5000-movie-dataset

3 Note that in both knowledge transfer approaches, item embeddings are randomly initialized on the target domain, since items are not shared between domains.

4
https://github.com/graytowne/caser_pytorch

5
https://github.com/jaywonchung/BERT4Rec-VAE-Pytorch

6
https://github.com/artem-oppermann/Deep-Autoencoders-For-Collaborative-Filtering

7 The observations are supported by the statistical significance test on the experiment results with p<0.05.
